# Association of the *Streptococcus bovis/Streptococcus equinus* Complex With Colorectal Neoplasia: A Systematic Review and Meta-analysis

**DOI:** 10.1093/ofid/ofad547

**Published:** 2023-10-31

**Authors:** Konstantinos Ouranos, Angeliki Gardikioti, Dimitra Rafailia Bakaloudi, Evangelia K Mylona, Fadi Shehadeh, Eleftherios Mylonakis

**Affiliations:** Department of Medicine, Houston Methodist Research Institute, Houston, Texas, USA; Herbert Irving Comprehensive Cancer Center, Columbia University Irving Medical Center, New York, New York, USA; Division of Oncology, Department of Medicine, University of Washington, Seattle, Washington, USA; Department of Medicine, Houston Methodist Research Institute, Houston, Texas, USA; Department of Medicine, Houston Methodist Research Institute, Houston, Texas, USA; School of Electrical and Computer Engineering, National Technical University of Athens, Athens, Greece; Department of Medicine, Houston Methodist Research Institute, Houston, Texas, USA; Weill Cornell Medicine, New York, New York, USA

**Keywords:** colorectal carcinoma, fecal carriage, invasive infection, serologic evidence, *Streptococcus bovis/Streptococcus equinus* complex

## Abstract

**Background:**

Invasive infection with *Streptococcus bovis/Streptococcus equinus* complex (SBSEC) bacteria is associated with underlying colorectal neoplasia. However, the link between intestinal or fecal colonization with SBSEC isolates or antibody responses to SBSEC members and colorectal cancer is not thoroughly investigated in the literature.

**Methods:**

We searched the PubMed, EMBASE, and Web of Science databases for case–control studies as well as retrospective or prospective cohort studies reporting an association between SBSEC bacteria and colorectal neoplasia.

**Results:**

We identified 22 studies (15 case–control and 7 cohort) that met our inclusion criteria. Among the cohort studies, patients with SBSEC bacteremia were 3.73 times more likely to have underlying colorectal cancer compared with individuals with no bacteremia (relative risk [RR], 3.73; 95% CI, 2.79–5.01), whereas the risk of underlying colorectal adenoma in patients with SBSEC bacteremia was not significantly increased (RR, 5.00; 95% CI, 0.83–30.03). In case–control studies, patients with colorectal cancer were 2.27 times more likely to have evidence of intestinal or fecal colonization with SBSEC isolates (odds ratio [OR], 2.27; 95% CI, 1.11–4.62) and immunoglobulin G (IgG) antibody responses to SBSEC antigens (OR, 2.27; 95% CI, 1.06–4.86) compared with controls. Patients with colorectal adenoma were not more likely to be colonized with SBSEC isolates compared with controls (OR, 1.12; 95% CI, 0.55–2.25).

**Conclusions:**

Apart from the well-established association of SBSEC bacteremia and underlying colorectal cancer, intestinal or fecal colonization with SBSEC isolates and IgG antibody responses to SBSEC antigens were higher in patients with colorectal cancer compared with controls. Neither bacteremia from SBSEC isolates nor colonization with SBSEC bacteria was associated with underlying colorectal adenoma.

Colorectal cancer is the third most common type of cancer and a leading cause of cancer-related mortality on a global scale [1]. Alteration in the gut microbiome homeostasis has been recognized as a major contributor to colorectal cancer development [2, 3]. The *Streptococcus bovis/Streptococcus equinus* complex (SBSEC) is a group of nonenterococcal group D streptococci that cause invasive disease, mainly bacteremia or infective endocarditis [4]. Initially, bacterial isolates belonging to SBSEC were phenotypically designated as *S. bovis* biotypes I, II/1, and II/2; the advent of molecular classification assays resulted in the reclassification of SBSEC isolates to *Streptococcus gallolyticus* subsp *gallolyticus* (I), *S. infantiarius* subsp *infantarius* (II/1), *S. infantiarius* subsp *coli* (II/1), and *S. gallolyticus* subsp *pasteurianus* (II/2) [5]. The association of SBSEC bacteremia with underlying colorectal neoplasia has been described in terms of pathogenesis [6, 7], and clinical evidence supporting a link between this complex and colorectal cancer is increasing [8], reinforcing the critical role of SBSEC in the detection of occult colorectal malignancy.

Although invasive SBSEC infection can signal the presence of underlying colorectal neoplasia, the detection of SBSEC isolates in asymptomatic patients has not been extensively studied to determine if it aids in the detection of colorectal cancer. The detection of SBSEC isolates in intestinal or fecal tissue samples in the general population ranges from 5% to 60%, with this variability being attributed to diagnostic modalities harnessed to detect SBSEC, and regional differences in terms of distribution of SBSEC bacteria in the population [9, 10]. In patients with hepatobiliary disorders, the detection rate of SBSEC ranges widely from 2% to 83%, depending on the SBSEC species under consideration [11–15]. In patients with colorectal cancer, colonization rates of up to 74% have been reported [16], with most data linking *S. gallolyticus* subsp *gallolyticus* with underlying colorectal malignancy, although associations with other SBSEC bacteria and colorectal cancer have also been described [17, 18]. The increased incidence of *S. gallolyticus* subsp *gallolyticus* colonization in colorectal cancer has been attributed to multiple mechanisms, including preferential attachment of the bacteria to exposed collagen fibers emerging from malignant colorectal tissue [18], increased bacterial competitiveness due to bile acid–induced toxin production that kills competing enterococci in the gastrointestinal tract [19], and utilization of glucose and its metabolites together with amino acids that confer a growth advantage for SBSEC bacteria [20]. Moreover, the presence of serum immunoglobulin G (IgG) antibodies against SBSEC antigens has been recently studied as a potential molecular marker for the presence of colorectal neoplasia in asymptomatic individuals. Several studies have demonstrated a positive association between seropositivity against SBSEC antigenic targets and underlying colorectal adenoma or carcinoma [21–24].

While the link between SBSEC bacteremia and colorectal cancer is well described [16, 25–27], the link between intestinal or fecal colonization with SBSEC isolates or serologic responses to SBSEC members and underlying colorectal neoplasia has not been extensively analyzed. In this systematic review and meta-analysis, we evaluated whether fecal or intestinal colonization with SBSEC isolates or evidence of IgG antibody responses to SBSEC antigens was associated with colorectal cancer risk. Furthermore, we aimed to reinforce the link between SBSEC bacteremia and underlying colorectal cancer.

## METHODS

### Approach

This systematic review and meta-analysis was performed in accordance with the Meta-Analysis of Observational Studies in Epidemiology (MOOSE) reporting guidelines (Supplementary Table 1) [28] and Preferred Reporting Items for Systematic Reviews and Meta-Analyses (PRISMA) statement checklist (Supplementary Table 2) [29].

### Data Sources

We searched the PubMed/MEDLINE, EMBASE, and Web of Science databases for literature in English (from database inception to June 30, 2023) using the following search terms: ((“*Streptococcus bovis*” OR *bovis* OR *gallolyticus* OR *infantarius* OR *pasteurianus* OR *coli* OR *lutetiensis*) AND (malignancy OR neoplasm OR cancer OR “colorectal cancer” OR “colonic neoplasia” OR “colonic adenocarcinoma” OR carriage OR “fecal carriage”)). Manual checking of reference lists of retrieved articles and reports, including relevant papers and meta-analyses, was performed to identify additional and potentially relevant articles. The *Rayyan* screening tool was used to create our database and screen the exported studies [30].

### Study Selection

Observational studies, including case–control and cohort studies, were assessed for inclusion in our analysis. For case–control studies, we defined cases as patients with a biopsy-proven diagnosis of colorectal neoplasia, defined as either colorectal adenoma or carcinoma, while controls were defined as individuals with no diagnosis of colorectal neoplasia. For retrospective or prospective cohort studies, the exposed group included patients aged >18 years with 1 of the following exposures of interest: (a) presence of SBSEC bacteremia and/or infective endocarditis, diagnosed via blood cultures (bacteremia), the Duke criteria, or the modified Duke criteria (infective endocarditis) [31]; (b) evidence of SBSEC colonization, defined as either culture of SBSEC isolates in colonic or fecal tissue samples or identification of SBSEC DNA through detection of the *sodA* gene via polymerase chain reaction (PCR) or in situ hybridization in colonic or fecal tissue samples; or (c) presence of IgG antibodies against the SBSEC cell wall and pili protein antigens in the serum (detailed information on diagnostic assays, antigenic targets, and cutoff values for IgG antibody positivity are presented in Supplementary Table 3). The control group included individuals with no evidence of SBSEC bacteremia, colonization, or serum IgG antibodies against SBSEC antigens.

The genotypic designation of the SBSEC was used to report associations between SBSEC and colorectal neoplasia, whenever available.

### Study Outcomes

For case–control studies, we examined the odds of intestinal or fecal colonization with SBSEC in patients with colorectal cancer or adenoma, as well as serologic responses against *S. gallolyticus* subsp *gallolyticus* antigens in patients with colorectal cancer compared with individuals with no diagnosis of colorectal neoplasia. For cohort studies, we examined the risk of colorectal cancer or adenoma in patients with SBSEC bacteremia (with or without concurrent infective endocarditis) compared with patients without bacteremia.

### Data Extraction and Quality Assessment

Two reviewers (K.O. and A.G.) independently determined study eligibility by screening titles and abstracts and performing full-text reviews of selected studies. Potential disagreements were resolved by a third reviewer (D.B.). Extractable data included baseline patient characteristics, methods used to diagnose bacteremia or colonization, SBSEC phenotypic and/or genotypic classification, primary and secondary outcomes, and information related to study quality.

Two reviewers (K.O. and A.G.) assessed the quality of the included studies using the Newcastle-Ottawa-Scale (NOS) [32], a tool developed for quality assessment of nonrandomized studies. The NOS checklist is a 9-point scale that involves the appraisal of methodological issues and their reporting. The scoring system encompasses 3 major domains (participant selection, group comparability, and ascertainment of exposure); scores range from 0 to 9, with scores ≥7 indicating high-quality studies.

### Data Synthesis and Analysis

For data analysis, we used Stata, version 17 (Stata Corporation, College Station, TX, USA). We performed a random-effects meta-analysis using the restricted maximum likelihood method [33] to estimate the risk of colorectal neoplasia in patients with SBSEC bacteremia compared with patients with no evidence of invasive infection. We also calculated the odds of intestinal or fecal colonization with SBSEC in patients with colorectal cancer or adenoma, as well as serum IgG antibody responses against *S. gallolyticus* subsp *gallolyticus* antigens in patients with colorectal cancer compared with individuals with no diagnosis of colorectal neoplasia. We reported odds ratios (ORs) and relative risks (RRs) with 95% confidence intervals. We estimated heterogeneity using the *I*^2^ statistic, with *I*^2^ values of 25%, 50%, and 75% representing low, moderate, and high heterogeneity, respectively [34]. We set statistical significance at α = .05. We used the Egger test to evaluate for publication bias and to assess small study effects [35].

We also conducted a subgroup analysis according to the genotypic classification of SBSEC to calculate the risk of colorectal cancer in patients with *S. gallolyticus* subsp *gallolyticus* bacteremia compared with individuals without bacteremia, as well as the odds of intestinal or fecal colonization with *S. gallolyticus* subsp *gallolyticus* in patients with colorectal cancer compared with controls.

## RESULTS

### Search Results

**Figure 1.**
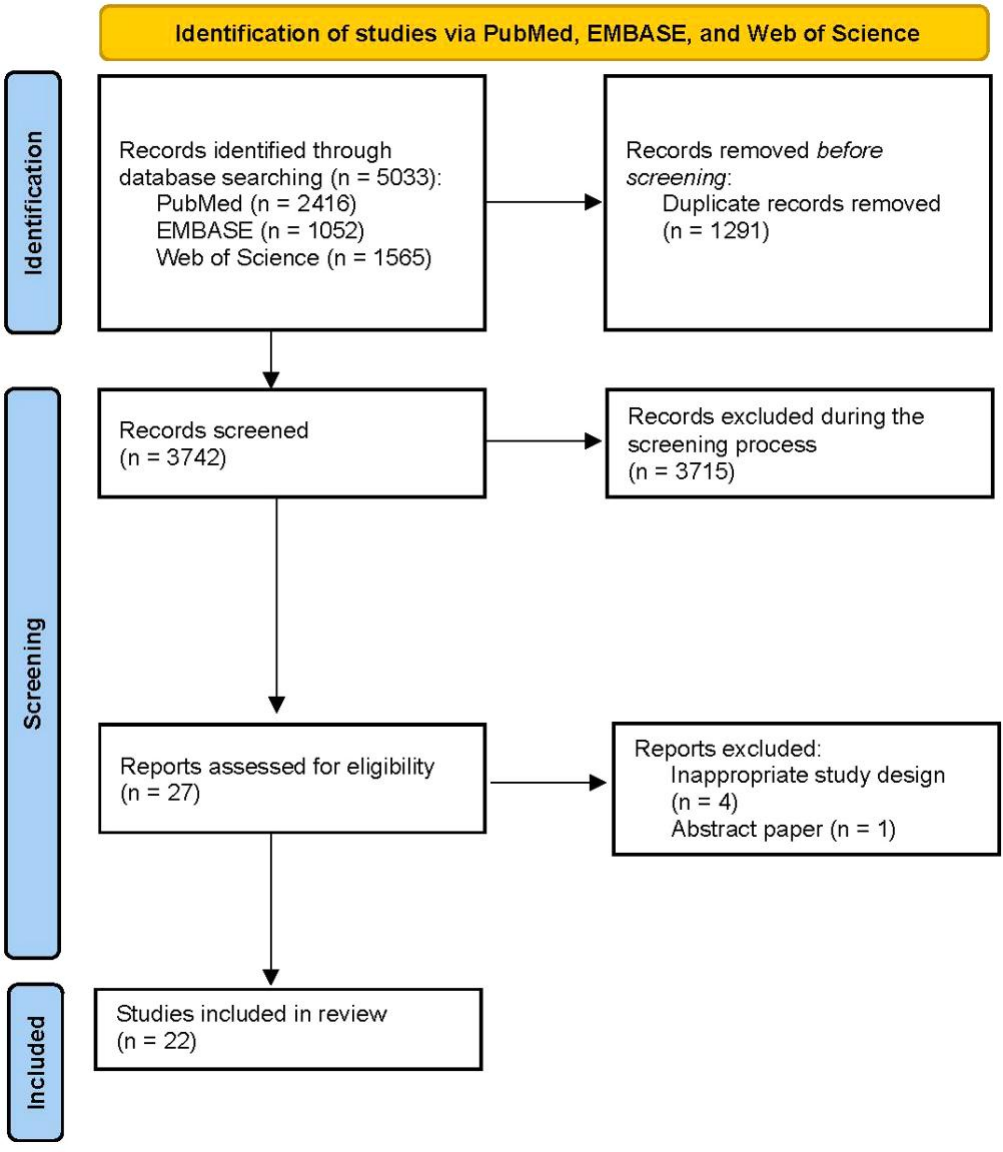
Flow diagram for the selection of studies included in the systematic review and meta-analysis.

We found 5033 studies from our literature search in PubMed, EMBASE, and Web of Science. After the removal of duplicates (n = 1291), we examined 3742 studies for eligibility. We excluded 3715 studies based on title and abstract assessment and found 22 studies that met our eligibility criteria (study schema detailed in Figure 1).

### Study Characteristics and Outcomes

From our analysis, we found 15 case–control [21–23, 36–47] and 7 (6 retrospective [48–53] and 1 prospective [54]) cohort studies. Table 1 summarizes the baseline characteristics of the case–control studies, and Table 2 shows the baseline characteristics of the cohort studies included in the analysis.

**Table 1.** Characteristics of Case–Control Studies Included in the Analysis

Study Author	Study Year	Study Population, No.	Age, Mean ± SD, y	Sex (Male)	Assessment	Site of SBSEC Identification	SBSEC Detection Method	SBSEC Genotypic and/or Phenotypic Classification^b^
Potter et al. [44]	1998	Colorectal cancer (19)	NA	9	Carriage^a^	Stool	Culture	*S. bovis* biotype II
		Controls (23)	NA	11				
Abdulamir et al. [37]	2009	Colorectal cancer (50)	57.1	27	Serologic evidence	Serum	IgG antibody detection to *S. gallolyticus* subsp *gallolyticus* cell wall antigens	*S. gallolyticus* subsp *gallolyticus*
		Colorectal adenoma (14)	57.1	27				
		Controls (30)	53.5	NA				
Abdulamir et al. [36]	2010	Colorectal cancer (91)	58 ± 7.4	40	Carriage^a^	Stool, mucus layer of colorectum, colorectal tissue	Culture, PCR assay of *sodA* gene–specific primer, *sodA* probe–based in situ hybridization assay	*S. gallolyticus* subsp *gallolyticus*
		Controls (50)	57.4 ± 4.7	27				
Rahimkhani et al. [45]	2010	Colorectal cancer (30)	56 ± 17.1	23	Carriage^a^	Stool	Culture	SBSEC
		Controls (30)	50.7 ± 16.7	20				
Vilardell et al. [47]	2021	Colorectal cancer (8)	68 ± 23	7	Bacteremia/infective endocarditis	Blood	Culture	*S. gallolyticus* subsp *gallolyticus*
		Colorectal adenoma (14)	69 ± 13	9				
		Controls (49)	65 ± 18	35				
Perichon et al. [43]	2022	Colorectal cancer (81)	64.1	53	Carriage^a^	Stool	PCR assay of *sodA1*/*sodA2* gene-specific primers	*S. gallolyticus* subsp *gallolyticus*
		Colorectal adenoma (23)	61.4	14				
		Controls (25)	63.7	13				
Mahmoudvand et al. [42]	2017	Colorectal cancer (70)	NA	NA	Carriage^a^	Colorectal tissue	PCR assay of *sod* gene–specific primer	*S. gallolyticus* subsp *gallolyticus*
		Colorectal adenoma (70)	NA	NA				
		Controls (70)	NA	NA				
Butt et al. [21]	2018	Colorectal cancer (485)	59	238	Serologic evidence	Serum	IgG antibody detection against Gallo0112A-B/272, 577, 748, 933, 1570, 1675, 2018, 2178, 2179	*S. gallolyticus* subsp *gallolyticus*
		Controls (485)	60	238				
Butt et al. [23]	2015	Colorectal cancer (576)	NA	285	Serologic evidence	Serum	IgG antibody detection against Gallo1569, 2039, 2178, 2179	*S. gallolyticus* subsp *gallolyticus*
		Controls (576)	NA	286				
Butt et al. [22]	2017	Colorectal cancer (318)	68	185	Serologic evidence	Serum	IgG antibody to Gallo2178, 2179	*S. gallolyticus* subsp *gallolyticus*
		Controls (228)	62	104				
Sheikh et al. [46]	2020	Colorectal cancer (22)	NA	16	Carriage^a^	Stool	Culture, PCR assay of *sodA* gene–specific primer, *sodA* probe–based in situ hybridization assay	*S. gallolyticus* subsp *gallolyticus*
		Controls (40)	NA	23				
Dubrow et al. [39]	1991	Colorectal cancer (9)	NA	NA	Carriage^a^	Stool	Culture	SBSEC
		Colorectal adenoma (38)	NA	NA				
		Controls (64)	NA	NA				
Klein et al. [41]	1977	CRC (75)	71	NA	Carriage^a^	Stool	Culture	SBSEC
		Controls (105)	48	NA				
Chirouze et al. [38]	2013	Colorectal cancer (4)	NA	NA	Carriage^a^	Stool	Culture	SBSEC
		Colorectal adenoma (45)	NA	NA				
		Controls (134)	NA	NA				
Genua et al. [40]	2023	Colorectal cancer (25)	66	12	Serologic evidence	Serum	IgG antibody detection against Gallo0112A, 0112B, 0272, 0577, 0748, 0933, 1570, 1675, 2018, 2178, 2179	*S. gallolyticus* subsp *gallolyticus*
		Colorectal adenoma (82)	63	46				
		Controls (100)	61	47				

Abbreviations: IgG, immunoglobulin G; NA, not applicable/available; PCR, polymerase chain reaction; SBSEC, *Streptococcus bovis/Streptococcus equinus* complex; *S. gallolyticus* subsp *gallolyticus*, *Streptococcus gallolyticus* subsp *gallolyticus*.

^a^Sites for SBSEC detection included fecal and/or colorectal tissue.

^b^When no phenotypic or genotypic classification was available, the general term SBSEC was used.

**Table 2.** Characteristics of Cohort Studies Included in the Analysis

Study Author	Study Year	Study Design	Exposure	Population, No.	Age, Mean ± SD, y	Sex (Male)	Site of SBSEC Identification	SBSEC Detection Method	SBSEC Genotypic and/or Phenotypic Classification^b^
Kwong et al. [50]	2018	Retrospective	Bacteremia	Exposed (662)	70 ± 38.4	NA	Blood	Culture	*S. gallolyticus* subsp *gallolyticus*
				Nonexposed (3310)	70.3 ± 38.3	NA			
Corrediora-Sanchez et al. [48]	2012	Retrospective	Bacteremia/infective endocarditis	Exposed (98)	66.2 ± 11.7	89	Blood	Culture	*S. gallolyticus* subsp *gallolyticus*
				Nonexposed (196)	66.3 ± 11.9	178			
Boltin et al. [54]	2015	Prospective	Carriage^a^	Exposed (15)	64.8 ± 8.9	10	Stool, colonic fluid, colonic tissue	Culture	SBSEC
				Nonexposed (103)	64.5 ± 9.3	65			
Shahara et al. [53]	2013	Retrospective	Bacteremia/infective endocarditis	Exposed (10)	67.9	5	Blood	Culture	SBSEC
				Nonexposed (200)	NA	NA			
Hoenn et al. [49]	1994	Retrospective	Bacteremia/infective endocarditis	Exposed (32)	61.3	27	Blood	Culture	SBSEC
				Nonexposed (64)	NA	NA			
Paritsky et al. [52]	2015	Retrospective	Carriage^a^	Exposed (49)	70.1	24	Colonic suction fluid	Culture	SBSEC
				Nonexposed (154)	60.1	77			
Laupland et al. [51]	2023	Retrospective	Bacteremia	Exposed (397)	NA	NA	Blood	Culture	*S. gallolyticus* subsp *gallolyticus, S. gallolyticus* subsp *pasteurianus, S. infantarius* subsp *infantarius, S. infantarius* subsp *lutetiensis*
				Nonexposed (84 339)	NA	NA			

Abbreviations: NA, not applicable/available; SBSEC, *Streptococcus bovis/Streptococcus equinus* complex; *S. gallolyticus* subsp *gallolyticus*, *Streptococcus gallolyticus* subsp *gallolyticus*; *S. gallolyticus* subsp *pasteurianus*, *Streptococcus gallolyticus* subsp *pasteurianus*; *S. infantarius* subsp *infantarius*, *Streptococcus infantarius* subsp *infantarius*; *S. infantarius* subsp *lutetiensis*, *Streptococcus infantarius* subsp *lutetiensis*.

^a^Sites for SBSEC detection included fecal and/or colorectal tissue.

^b^When no phenotypic or genotypic classification was available, the general term SBSEC was used.

### SBSEC Bacteremia and Underlying Colorectal Neoplasia

**Figure 2.**
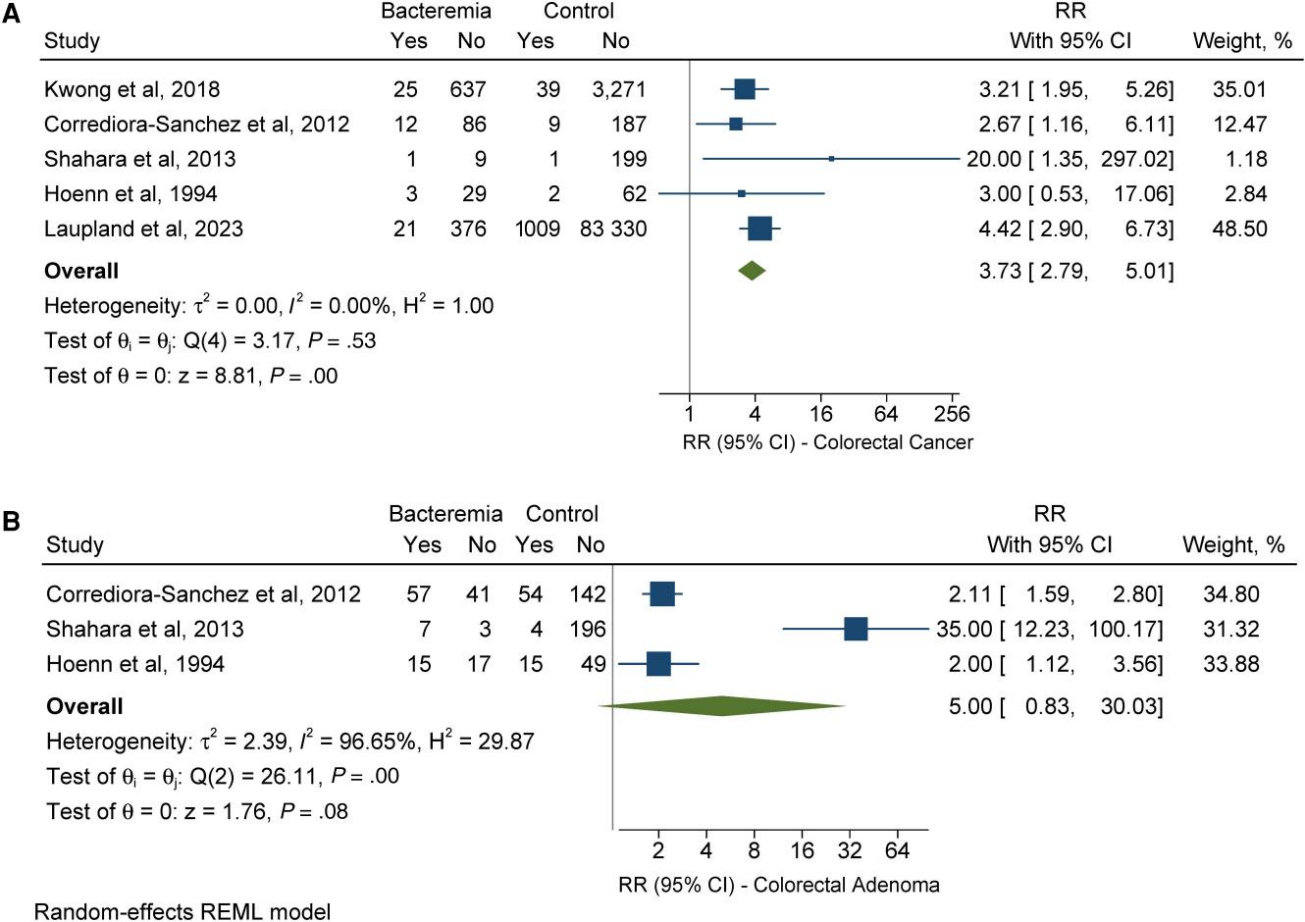
Individual and combined risk of colorectal neoplasia, either carcinoma (*A*) or adenoma (*B*), in patients with SBSEC bacteremia (with or without concomitant infective endocarditis) compared with individuals with no bacteremia diagnosis. The size of the squares is proportional to the weight of each study. Horizontal lines indicate the 95% CI of each study; diamond, the pooled estimate with 95% CI. Abbreviations: REML, restricted maximum likelihood; RR, relative risk; SBSEC, *Streptococcus bovis/Streptococcus equinus* complex.

Five cohort studies assessed the risk of underlying colorectal cancer diagnosis in patients with and without SBSEC bacteremia [48–51, 53]. Specifically, 62 out of 1199 (5.2%) and 1060 out of 88 109 (1.2%) patients with and without SBSEC bacteremia, respectively, had underlying colorectal cancer. Patients with SBSEC bacteremia were 3.73 times more likely to have an underlying diagnosis of colorectal cancer compared with patients with no diagnosis of bacteremia (RR, 3.73; 95% CI, 2.79–5.01; *I*^2^ = 0.00%) (Figure 2*A*).

In 3 cohort studies included [48, 49, 53], 79 out of 140 (56.4%) and 74 out of 460 (16.1%) patients with and without SBSEC bacteremia, respectively, had an underlying diagnosis of colorectal adenoma. Although colorectal adenoma was detected in 56.4% of patients with SBSEC bacteremia, compared with 16.1% of patients without bacteremia, the difference in the detection rate was not statistically significant (RR, 5.00; 95% CI, 0.83–30.03; *I*^2^ = 96.65%) (Figure 2*B*).

Genotypic classification of the SBSEC was available in 3 cohort studies [48, 50, 51], in which 26 out of 417 (6.2%) and 627 out of 54 541 (1.1%) patients with and without *S. gallolyticus* subsp *gallolyticus* bacteremia, respectively, had underlying colorectal cancer. Patients with *S. gallolyticus* subsp *gallolyticus* bacteremia were 3.66 times more likely to have a diagnosis of colorectal neoplasia compared with controls (RR, 3.66; 95% CI, 2.25–5.93; *I*^2^ = 0.00%) (Supplementary Figure 1).

### Intestinal or Fecal Colonization With SBSEC and Underlying Colorectal Neoplasia

**Figure 3.**
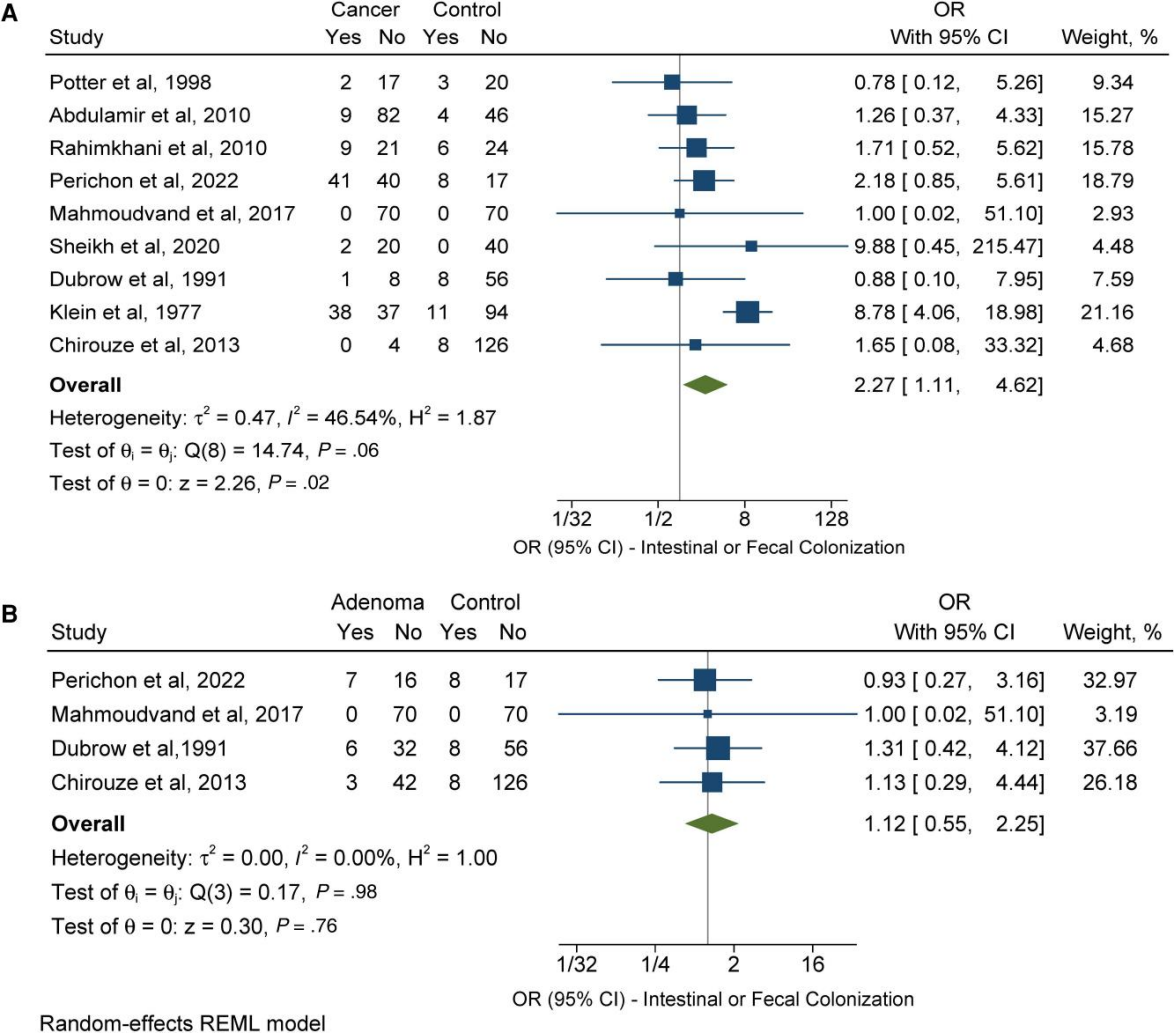
Individual and combined odds of colonization with SBSEC isolates in fecal or intestinal tissue in patients with colorectal neoplasia, either carcinoma (*A*) or adenoma (*B*), compared with individuals with no colorectal neoplasia diagnosis. The size of the squares is proportional to the weight of each study. Horizontal lines indicate the 95% CI of each study; diamond, the pooled estimate with 95% CI. Abbreviations: OR, odds ratio; REML, restricted maximum likelihood; SBSEC, *Streptococcus bovis/Streptococcus equinus* complex.

Isolation of SBSEC bacteria from intestinal or fecal tissue samples was performed in 9 case–control studies [36, 38, 39, 41–46]. Among them, 7 studies [38, 39, 41, 43–46] used stool cultures to detect SBSEC isolates, 3 studies [36, 43, 46] identified SBSEC bacteria through detection of the *sodA* gene via PCR, 2 studies [36, 46] used *sodA* probe-based in situ hybridization assay in fecal samples, and 2 studies [36, 42] used PCR in colorectal tissue for SBSEC detection. Out of these studies, 102 out of 401 (25.4%) patients with colorectal cancer and 48 out of 541 (8.9%) controls had evidence of SBSEC bacteria isolated from intestinal or fecal tissue samples. Patients with colorectal cancer were 2.27 times more likely to have evidence of colonization with SBSEC isolates compared with individuals with no colorectal cancer diagnosis (OR, 2.27; 95% CI, 1.11–4.62; *I*^2^ = 46.54%) (Figure 3*A*). Egger's test showed no evidence of publication bias or small study effects (bias = −0.94; *P* = .26).

Genotypic classification of the SBSEC was available in 4 case–control studies [36, 42, 43, 46], in which 52 out of 264 (19.7%) patients with colorectal cancer and 12 out of 185 (6.5%) controls had evidence of *S. gallolyticus* subsp *gallolyticus* colonization in intestinal or fecal tissue samples. However, patients with *S. gallolyticus* subsp *gallolyticus* colonization were not more likely to have an underlying diagnosis of colorectal cancer compared with controls (OR, 1.91; 95% CI, 0.93–3.92; *I*^2^ = 0.00%) (Supplementary Figure 2).

Moreover, in 4 case–control studies included [38, 39, 42, 43], 16 out of 176 (9.1%) patients with colorectal adenoma and 24 out of 293 (8.2%) controls had evidence of colonization with SBSEC isolates. Patients with colorectal adenoma, however, were not more likely to have evidence of SBSEC isolates in colonic or fecal tissue samples compared with controls (OR, 1.12; 95% CI, 0.55–2.25; *I*^2^ = 0.00%) (Figure 3*B*).

### IgG Antibody Responses to SBSEC Antigens and Underlying Colorectal Neoplasia

**Figure 4.**
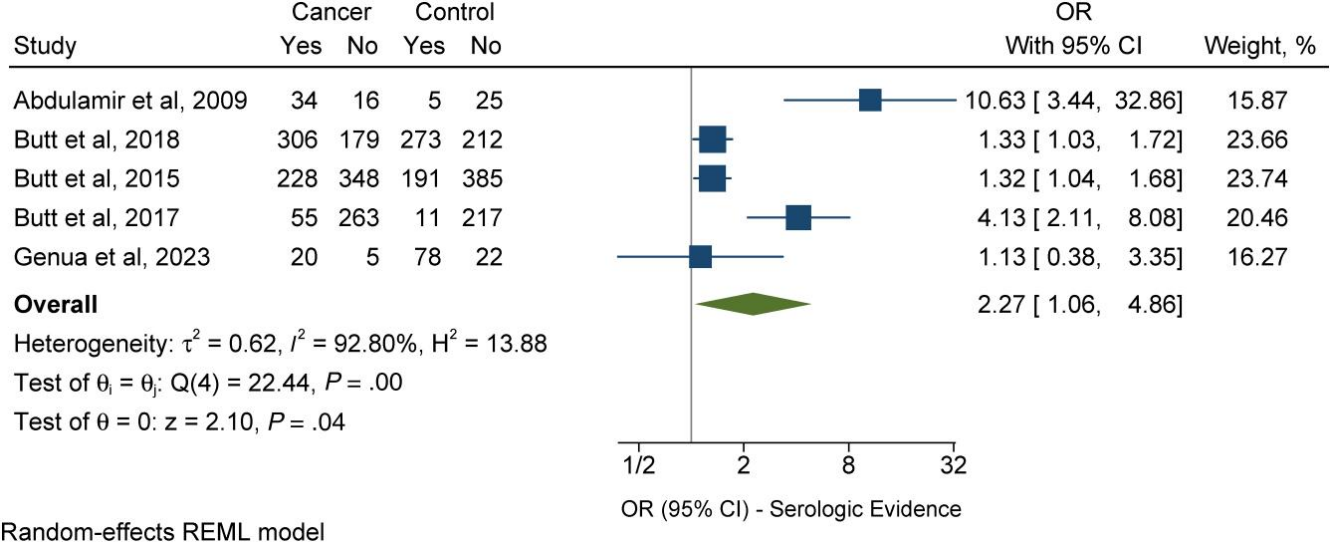
Individual and combined odds of immunoglobulin G antibody responses to *S. gallolyticus* subsp *gallolyticus* antigens in patients with colorectal cancer compared with individuals with no colorectal cancer diagnosis. The size of the squares is proportional to the weight of each study. Horizontal lines indicate the 95% CI of each study; diamond, the pooled estimate with 95% CI. Abbreviations: OR, odds ratio; REML, restricted maximum likelihood; *S. gallolyticus* subsp *gallolyticus*, *Streptococcus gallolyticus* subsp *gallolyticus*.

Five case–control studies assessed IgG antibody responses against cell wall and pili protein antigens expressed by *S. gallolyticus* subsp *gallolyticus* in patients with colorectal cancer compared with controls [21–23, 37, 40]. Out of these studies, 643 out of 1454 (44.2%) patients with colorectal cancer and 558 out of 1419 (39.3%) controls had IgG antibodies detected against *S. gallolyticus* subsp *gallolyticus* cell wall [37] and pili protein [21–23, 40] antigens. Patients with colorectal cancer were 2.27 times more likely to have serologic responses against *S. gallolyticus* subsp *gallolyticus* antigens compared with controls (OR, 2.27; 95% CI, 1.06–4.86; *I*^2^ = 92.80%) (Figure 4).

### Quality Assessment of the Studies Included in the Analysis

Supplementary Table 4 shows the overall study quality assessment for the case–control studies included in the analysis, and Supplementary Table 5 summarizes the overall study quality assessment for the cohort studies included in the analysis, according to the NOS checklist. The inclusion of studies classified as high quality with regards to participant selection, group comparability, and outcome assessment helps strengthen our meta-analysis results. Overall, we classified 11 case–control studies as high quality (NOS ≥7) [21–23, 36–38, 40, 42, 43, 45, 46] and 4 studies as fair quality (NOS 5–6) [39, 41, 44, 47]. Regarding cohort studies, we classified 3 studies as high quality (NOS ≥7) [50, 52, 54] and 4 studies as fair quality (NOS 5–6) [39, 41, 44, 47].

### Sensitivity Analysis According to NOS Quality Assessment

We repeated the main analysis by including only the high-quality studies (NOS ≥7) to explore the robustness of our findings and ensure that aspects regarding study design and outcome assessment would not interfere with the interpretation of our meta-analysis results. After excluding fair-quality studies, a subgroup analysis was available only for case–control studies estimating the odds of colonization with SBSEC isolates in patients with colorectal neoplasia compared with controls. A sensitivity analysis was not carried out for evaluating the association between SBSEC bacteremia or IgG antibody responses to SBSEC antigens and colorectal neoplasia due to an insufficient number of high-quality studies.

We included 6 high-quality studies reporting information on patients with colorectal cancer [36, 38, 42, 43, 45, 46], and the sensitivity analysis showed that patients with colorectal cancer were 1.85 times more likely to have evidence of colonization with SBSEC isolates in fecal or intestinal tissues compared with controls (OR, 1.85; 95% CI, 1.01–3.38; *I*^2^ = 0.00%) (Supplementary Figure 3). Next, of the 3 studies that had information about individuals with colorectal adenoma [38, 42, 43], colonization with SBSEC isolates in fecal or intestinal tissues was not observed, with higher odds in patients with colorectal adenoma compared with controls (OR, 1.01; 95% CI, 0.42–2.46; *I*^2^ = 0.00%) (Supplementary Figure 4).

## DISCUSSION

In this systematic review and meta-analysis, we explored the association between SBSEC and colorectal neoplasia. We found that patients with colorectal cancer were 2.27 times more likely to be colonized by SBSEC bacteria or have IgG antibody responses to *S. gallolyticus* subsp *gallolyticus* antigens compared with controls. Also, patients with SBSEC bacteremia were 3.73 times more likely to have underlying colorectal cancer compared with controls. Interestingly, our analysis did not find an association between SBSEC bacteremia or colonization and underlying colorectal adenoma diagnosis.

In our analysis, SBSEC bacteremia, and especially *S. gallolyticus* subsp *gallolyticus* invasive infection, was associated with underlying colorectal cancer. Consistent with our findings, a previous meta-analysis, including 3 cohort studies with 140 patients with SBSEC bacteremia and 450 controls, concluded that colorectal cancer risk was almost 8 times higher in patients with SBSEC bacteremia [26]. A meta-analysis by Boleij et al. [55] summarized the results of 52 case reports and 31 case series with a total of 189 patients with *S. gallolyticus* subsp *gallolyticus* and 151 patients with *S. bovis* biotype II bacteremia. The authors found that patients with *S. gallolyticus* subsp *gallolyticus* bacteremia had a higher risk of colorectal cancer compared with patients with *S. bovis* biotype II bacteremia. However, our meta-analysis included case–control and cohort studies, and we excluded case reports and case series. Overall, only 1 study [49] that was included in the meta-analysis by Boleij et al. [55] was included in our analysis as well, with the rest of the studies included adding new information and consolidating the association between SBSEC and colorectal neoplasia. Taken together, our meta-analysis included observational studies that assessed the risk of colorectal neoplasia in patients with evidence of SBSEC bacteremia, colonization, and antibody responses to SBSEC antigens. In contrast, the study by Boleij et al. [55] restricted their evaluation to the association between SBSEC clinical infection and colorectal cancer, which could explain the differential findings.

Interestingly, we found that IgG antibody responses to *S. gallolyticus* subsp *gallolyticus* antigens were more common in patients with colorectal cancer than controls. However, the presence of such antibodies is not able to indicate acute, localized infection in the colon, and it is unknown whether antibody development for *S. gallolyticus* subsp *gallolyticus* antigens occurs only after bacterial entry into the bloodstream or even when the bacterium is still located in the colon [56]. Regardless of when antibody positivity occurs, documenting the time from antibody detection to colorectal cancer diagnosis may aid in a better understanding of the role of *S. gallolyticus* subsp *gallolyticus* in carcinogenesis. Colorectal cancer is the result of a sequence of events leading from normal tissue to adenoma development and then to carcinoma, which can last more than 10 years [57].

From the studies included in our analysis, Butt et al. prospectively assessed patients with and without IgG antibodies against *S. gallolyticus* subsp *gallolyticus* pili protein antigens for colorectal cancer development and found that serologic responses to *S. gallolyticus* subsp *gallolyticus* antigens were 1.36 times more common in patients who developed colorectal cancer, in whom the mean time from blood draw to cancer diagnosis was 3.4 years [21]. The latter observation suggests that carcinogenesis had already begun in some patients with antibodies against *S. gallolyticus* subsp *gallolyticus* antigens. Similarly, a large prospective study of 4063 patients with colorectal cancer found that cancer risk was associated with IgG antibody detection against *S. gallolyticus* subsp *gallolyticus* pilus proteins within 10 years of blood draw, but not beyond 10 years of antibody positivity. Individuals diagnosed within 10 years of blood draw were also more likely to have precursor lesions in their colon [56]. Taken together, these findings suggest that once IgG antibodies are detected in the serum, patients may already have colonic premalignant lesions. More prospective studies that delineate the natural history of these antibodies are required to establish whether seropositivity for *S. gallolyticus* subsp *gallolyticus* antigens can be used as a marker of underlying colorectal neoplasia.

Our results showed that patients with colorectal cancer, but not colorectal adenoma, were more likely to be colonized by SBSEC isolates compared with controls. Contrary to our findings, a previous meta-analysis assessing 515 patients with colorectal neoplasia (either adenoma or carcinoma) and 642 controls found that culture of SBSEC isolates from fecal specimens did not occur with greater frequency in patients with colorectal neoplasia compared with controls [26]. Also, Boltin et al. prospectively assessed, over a period of 17 years, 15 individuals with and 100 individuals without evidence of SBSEC bacteria detected via culture in stool or colonic suction fluid and did not find a difference in colorectal cancer diagnosis between the groups [54]. The difference in results between our analysis and the literature may be attributed to the fact that we included a sufficient number of patients colonized with SBSEC bacteria to detect a difference in colonization rates between patients with colorectal cancer and controls. Indeed, although only 1 [41] out of the 9 studies in our analysis found a significant association between SBSEC colonization and colorectal cancer, combining the results of the individual studies in our analysis found that patients with colorectal cancer were more likely to be colonized with SBSEC bacteria compared with controls.

Interestingly, our subgroup analysis found that colonization with *S. gallolyticus* subsp *gallolyticus* was not associated with underlying colorectal cancer diagnosis. Out of all members of the SBSEC, *S. gallolyticus* subsp *gallolyticus* has been described to adhere to malignant colorectal tissue with higher affinity than other SBSEC bacteria due to the unique property of its pili proteins binding exposed collagen fibers emerging from the basal lamina of malignant tissue [55, 58, 59]. Moreover, Taddese et al. [60] found that *S. gallolyticus* subsp *gallolyticus* promotes tumorigenesis by modifying the ability of intestinal and cancerous cells to detoxify dietary components, thus allowing for DNA damage to accumulate, eventually increasing cancer risk [61, 62]. Kumar et al. [16] also demonstrated that *S. gallolyticus* subsp *gallolyticus* enhanced cellular proliferation by upregulating beta-catenin, a pivotal molecule in colon tumorigenesis [63], while Oehmcke-Hecht et al. [64] found that S. *gallolyticus* subsp *gallolyticus* degraded tannic acid, which is a substance known to have cytotoxic effects against colorectal cancer cells [65]. Failure to reveal a statistically significant association between *S. gallolyticus* subsp *gallolyticus* colonization and colorectal cancer can be explained by the small sample size used in our subgroup analysis or the involvement of other SBSEC bacteria in colorectal cancer development. Associations between SBSEC bacteria other than *S. gallolyticus* subsp *gallolyticus* and colorectal neoplasia stem from case reports and case series [11–13, 17, 51]. Also, there are limited mechanistic studies available to provide plausible mechanisms by which SBSEC members other than *S. gallolyticus* subsp *gallolyticus* may preferentially colonize colorectal tissue [58]. As a result, we highlight the importance of conducting studies with larger numbers of participants, in whom genotypic classification of SBSEC will occur, to establish the association between colonization with different SBSEC isolates and colorectal cancer.

Regarding the limitations of our analysis, only 2 studies reported underlying patient comorbidities [48, 54] and only 3 studies had information about family history of colorectal cancer [22, 23, 54], so we could not assess whether these variables confound the relationship between the SBSEC and colorectal cancer. Genotypic classification of the SBSEC was not available in 9 studies included in the analysis [38, 39, 41, 44, 45, 49, 52–54], and the remaining studies, with 1 exception [51], had information only about *S. gallolyticus* subsp *gallolyticus,* so comparison of the different subspecies with regards to risk of colorectal neoplasia was not possible. Next, colonization with SBSEC isolates was assessed via fecal culture evaluation in 7 studies [38, 39, 41, 43–46], and only 2 studies [36, 42] examined colorectal tissue for SBSEC detection. As such, we can only conclude that colonization with SBSEC isolates was higher in patients with colorectal cancer compared with controls, but we cannot comment on whether SBSEC bacteria isolated from fecal or colonic tissue samples were more closely linked to colorectal cancer. Finally, serologic responses were examined against various *S. gallolyticus* subsp *gallolyticus* antigens, and we could not perform separate analyses for specific antigenic targets due to insufficient data.

In conclusion, our analysis reinforces results from previous studies that link SBSEC infection or colonization with enhanced colorectal cancer risk and associate serologic responses to *S. gallolyticus* subsp *gallolyticus* antigens with underlying colorectal neoplasia. Strengthening of these observations is necessary as SBSEC detection in the blood or isolation from fecal or colonic tissue can be used as a marker of underlying malignancy. Although evidence about the role of SBSEC in colorectal adenoma development is available in experimental and observational studies [48, 49, 53], it remains to be seen whether detection of premalignant lesions can be achieved through isolation of SBSEC bacteria in the blood or colorectal tissue. Well-designed prospective studies on the role of antibody responses to *S. gallolyticus* subsp *gallolyticus* antigens can also provide us with immunological markers for early detection of colorectal cancer.

## Supplementary Material

ofad547_Supplementary_Data

## Supplementary Data

Supplementary materials are available at *Open Forum Infectious Diseases* online. Consisting of data provided by the authors to benefit the reader, the posted materials are not copyedited and are the sole responsibility of the authors, so questions or comments should be addressed to the corresponding author.
